# Supplementary figures and images for: Plasmodium berghei Kinesin-5 Associates With the Spindle Apparatus During Cell Division and Is Important for Efficient Production of Infectious Sporozoites
Source: Front Cell Infect Microbiol. 2020 Oct 14;10:583812. doi: 10.3389/fcimb.2020.583812 (PMC7591757; doi:10.3389/fcimb.2020.583812)

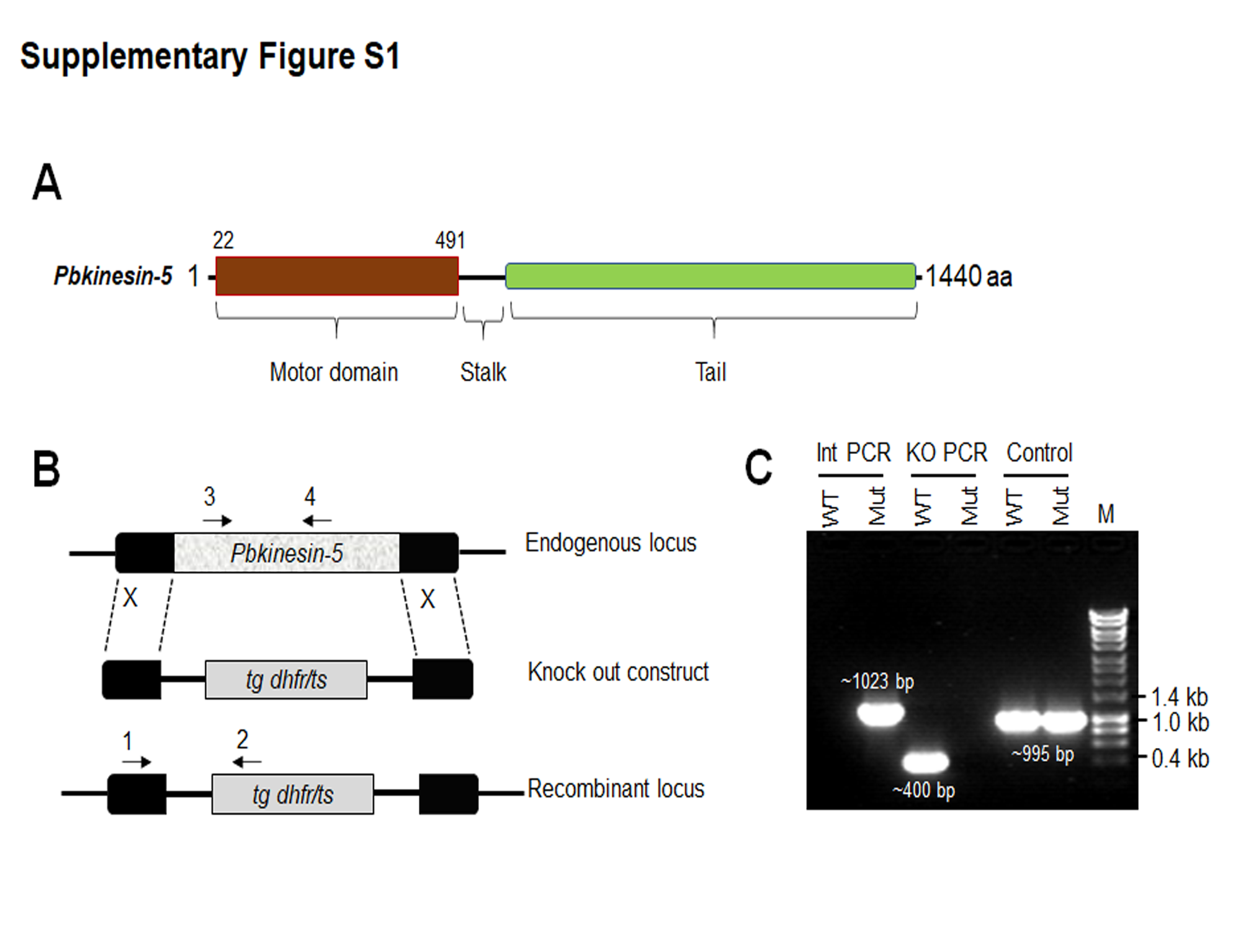

Supplement: Supplementary Figure 1 — Generation and genotype analysis of Δkinesin-5 parasites. (A) Schematic of Plasmodium berghei kinesin-5 protein (1-1440 aa) showing different domains. Approximate length of different domains is indicated. aa; amino acids. (B) Schematic representation of the endogenous kinesin-5 locus, the targeting gene deletion construct and the recombined kinesin-5 locus following double homologous recombination. (B) Integration PCR showing correct integration with expected size of bands and deletion of kinesin-5 gene from knockout (mut). [file Image_1.tif]

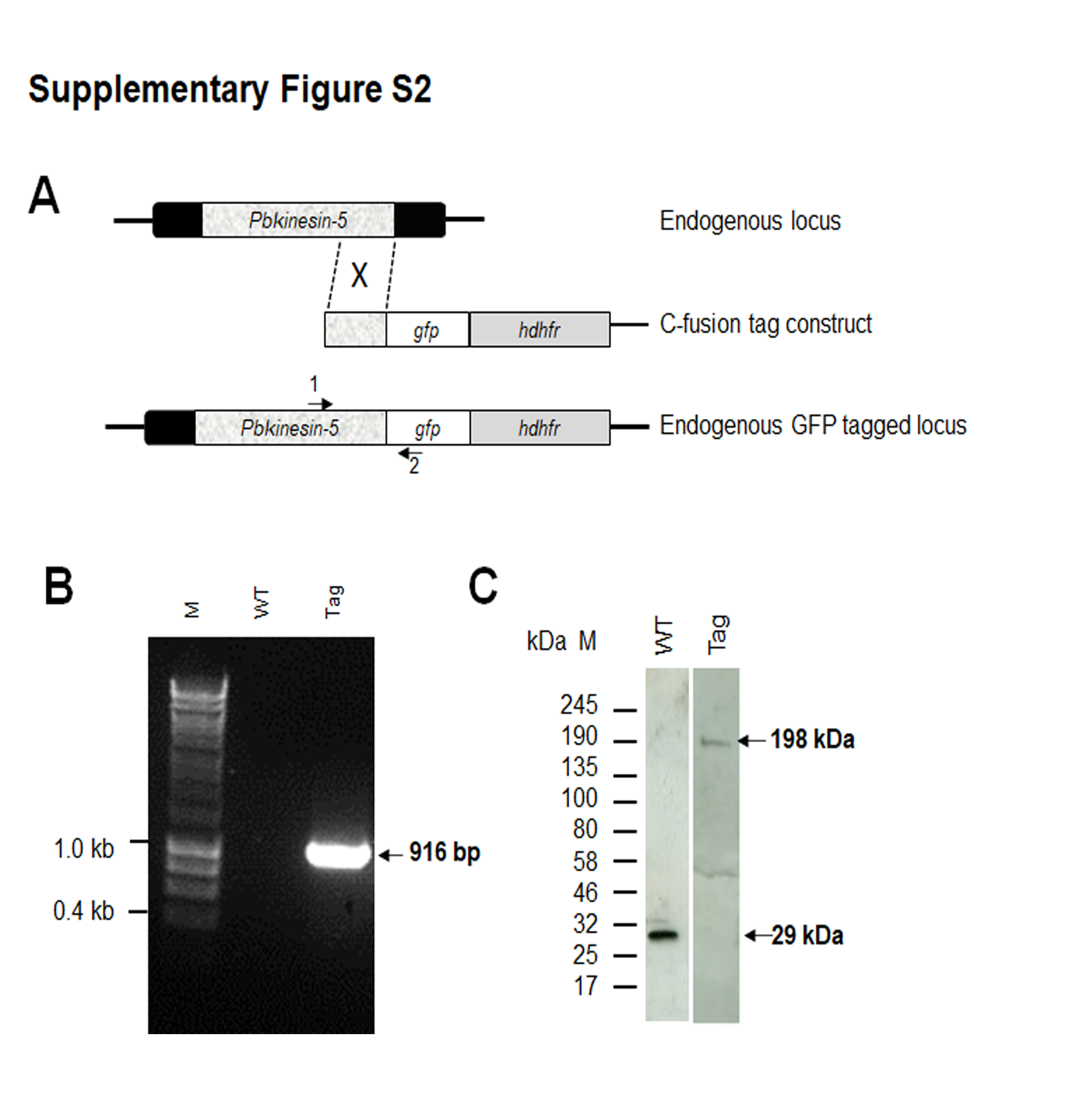

Supplement: Supplementary Figure 2 — Generation and genotypic analysis of kinesin-5-GFP parasites. (A) Schematic representation for 3’-tagging of kinesin-5 gene with green fluorescent protein (GFP) sequence via single homologous recombination. (B) Integration PCR showing correct integration of tagging construct. (C) Western blot showing expected size of kinesin-5-GFP protein. [file Image_2.tif]

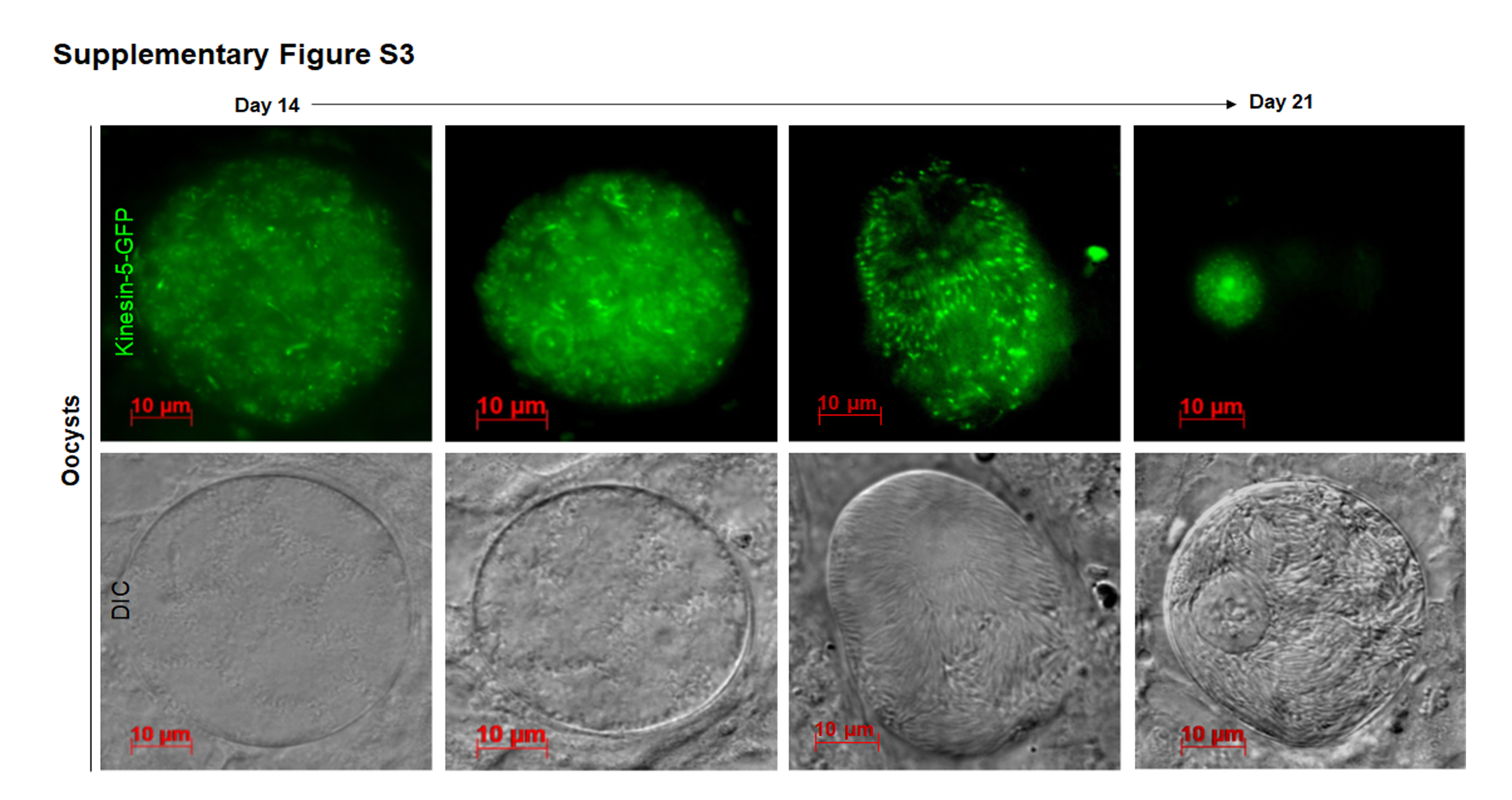

Supplement: Supplementary Figure 3 — Expression and localization of kinesin-5 during sporulation in oocysts. Live cell imaging showing the kinesin-5-GFP fluorescence in a developing oocyst between day 14 (sporulation starts) and day 21 (completely sporulated) after infection. [file Image_3.tif]
